# Exploring the barriers and facilitators of dietary self-care for type 2 diabetes: a qualitative study in Ghana

**DOI:** 10.15171/hpp.2019.31

**Published:** 2019-08-06

**Authors:** Martin Hushie

**Affiliations:** University for Development Studies, School of Allied Health Sciences Department of Behavioural Sciences, P. O. Box 1883, Tamale, N/R Ghana

**Keywords:** Type 2 diabetes mellitus, Diet, Self-care, Ghana, Health promotion, Health education, Social support

## Abstract

**Background:** There is an increasing prevalence of type 2 diabetes (T2D) globally and countries in Sub Sahara Africa, such as Ghana are contending with the epidemic. The main objective ofthis study was to explore the barriers and facilitators of T2D self-care as perceived by patients and health providers (HPs) in Ghana.

**Methods: ** A maximum variation sample of 33 adult patients with a range of demographic features, diabetic conditions and self-care regimens and 3 providers were purposely selected from the specialist diabetes clinic of a private hospital in Accra, Ghana. Data were collected using in-depth interviews, which were recorded and transcribed; and non-participant observational field notes-that were analyzed thematically through directed content analysis.

**Results: ** The findings reveal that T2D adult patients face many inter-related challenges to diabetes self-care, than enabling factors that fell into four major domains:1) the counselling process and context (patients missing follow-up appointments, unacceptance of diagnosis); 2)recommended food and diet regimens (changing habitual diets, dislike and confusion about recommended diets); 3) social aspects (social functions interfering with dietary regimens, family members diverting patient from dietary goals) and 4) fears (non-disclosure to family member/pretense of being well).

** Conclusion:** Integrated self-management interventions are needed to address these barriers, including tailoring dietary education to patients’ specific needs, guiding patients on how tomanage diet during social occasions and among family members; and as well, providing mental health support. Future research should focus on T2D self-care behaviours and practices outside the clinic, including home, work and shopping environments.

## Introduction

Diabetes is a serious chronic, non-communicable disease whose prevalence has been on the ascendancy worldwide. In 2017, an estimated 15.5 (9.8-27.8) million adults were living with the disease in sub-Saharan Africa (SSA) with a regional prevalence of ~ 6%. By 2045, this number is expected to increase by 162.5%, with an estimated 40.7 million suffering from type 2 diabetes (T2D).^1^ The growing prevalence of diabetes in SSA, which is expected to outstrip all other global regions has been linked to behavioural factors, including ageing populations, increasing levels of obesity, physical inactivity, poor dietary habits as well as limited health and social care resources for tackling the disease.^2-4^

In Ghana, the number of adults estimated to be living with diabetes in 2017 was 518.4 thousand with a country prevalence of ~ 3.6% and associated health care costs of US$55.2 million. This number is expected to increase to 1426.3 million by 2045 with costs rising to US$132.9 million.^1^ Studies in the general population have estimated a diabetes prevalence of between 6% and 9% among the adult population in urban areas with rural areas experiencing a lower prvalence.^5^ Factors such as an ageing population and improved socio-economic status; as well as interactions between acculturation, urbanization, and genetic disposition have all been cited as contributing towards this prevalence.^1,6,7^

Although the past few decades have witnessed the development of novel drugs that play a key role in the clinical management of T2D, research has suggested that self-care behaviours involving the patient working in collaboration with health care providers to engage in health promotion activities such as diet, physical activity are also important.^8,9^ Engaging in diabetes self-care is often recognized as the gateway to maintaining the health of diabetes patients, as effective self-management is not only useful for improving glycemic control, but it may also prevent diabetes-related complications, hospitalization and mortality.^10,11^ Despite health professionals’ role in facilitating and supporting health education and promotion activities for diabetes self-care, the responsibility of inducing lifestyle changes for maintaining health ultimately rests with the patient and can be challenging to achieve in practice.

Understanding the factors that create barriers or facilitate diabetes self-care is important in developing appropriate educational materials and prioritizing interventions that empower individuals to better take care of themselves. A large number of qualitative studies have examined barriers and facilitators of diabetes self-care in high income countries.^12-14^ with few studies exploring such phenomena in SSA countries.^15,16^ Although some studies in Ghana have examined the level of adherence and factors associated with self-care behaviors in T2D patients and found patients’ demographic characteristics (e.g., age, gender, education, and religion) diet and body weight as significant shaping influences on behaviours,^17,18^ much less attention has focused on the day-to-day challenges patients face and the facilitating factors in the management of the disease. In the last few years, controlling chronic, non-communicable diseases including diabetes has increasingly become a priority area in national health polices of many of many SSA countries^19^ and following this trend, Ghana’s Ministry of Health (MOH) has since 2012, developed a non-communicable disease policy and established centres that provide epidemiological evidence to guide national diabetes strategies.^5^ Against this background of increased policy interest in diabetes care and control, not much is known about patient’s and health provider’s (HP’s) views about lifestyle self-management. Such research is important in order to identify areas that require further improvement regarding patient counselling for promoting better health outcomes in diabetic patients.

The aim of this qualitative study was to explore the barriers and facilitators of T2D self-management from the perspectives of patients and HPs in Ghana.

## Materials and Methods

### Setting

The study setting was the specialist diabetes clinic of a private hospital located in Accra, the capital city of Ghana.

### Study design

A qualitative study with directed content analysis approach^20^ was used to explore the barriers and facilitators of T2D self-management as perceived by patients and HPs.

### Study participants 

Purposeful sampling to achieve maximum variation^21^ were used to select 33 adult patients with a range of demographic features (e.g., gender, ethnicity, educational and income levels); diabetic conditions and self-care regimens; and 3 HPs with educational backgrounds in dietetics and nutrition, providing treatment and patient education services at the diabetes clinic. Inclusion criteria for patients required that: 1) they were above 40 years, as diabetes prevalence tends to be higher among adults beyond this age^1^; 2) had an established diagnosis of T2D for at least 1 year; and 3) had been receiving diabetes care from the same clinic during this period (as it may take some time for the patient to adjust to a new routine which includes dietary regimens or to realize the barriers that do not permit them to adopt such regimens). Patients were excluded if they had multiple complications, were pregnant or breastfeeding or had a recent hospital admission- circumstances that could interfere with a regular lifestyle pattern for diabetes self-care. Patients who fulfilled the necessary characteristics required for the maximum variation sample were invited to participate. In all, 41 patients were approached, however only 33 agreed to be interviewed. The remaining 8, declined participation for lack of time and discomfort with discussing issues about diabetes self-care.

### Data collection 

**Figure 1:**
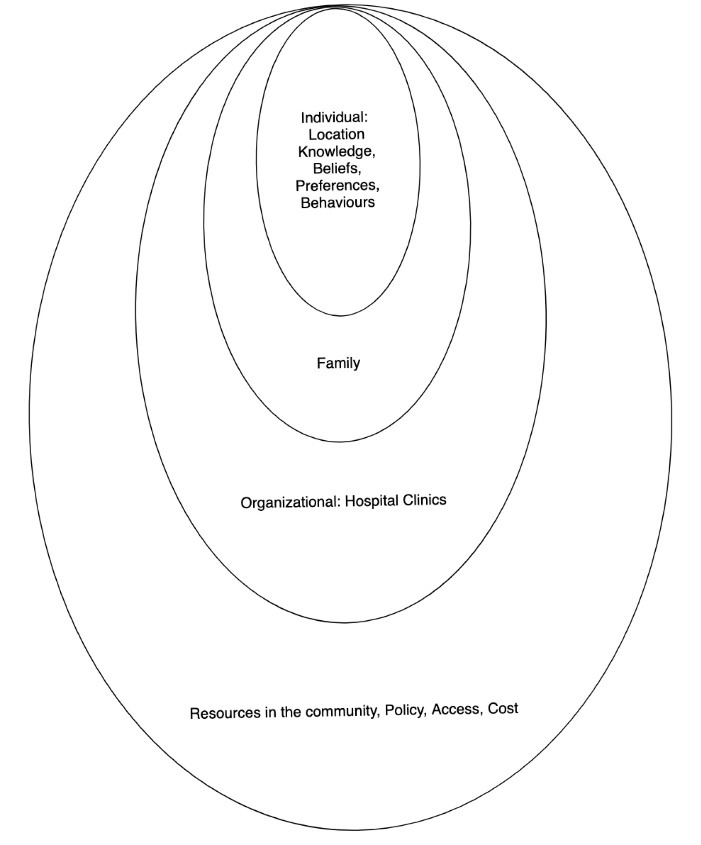
Socio-ecological behavioural model.

Data collection took place over a period of six months in 2015. Two major methods-non-participant observation and in-depth interviews were triangulated to obtain more reliable information from participants. Observation of patients and HPs took place over a period of 8 weeks, lasting over 1400 hours using an observation checklist and field note-taking, which were later expanded to provide more descriptive accounts of the events observed. The data gathered focused on the counselling process, including patients’ time of arrival at the out-patient’s department, waiting times, the nature of care provided and prevailing atmosphere. This technique was found valuable because it provided the opportunity to understand the variety of information given to patients regarding diet, how dietary education took place in terms of presentation, language and models; and patients’ reactions to this.^22^ In-depth semi-structured interviews were also conducted with HPs and patients based on a topic guide using the socio-ecological model (SEM),^23^ which holds that certain behaviors of individuals might be shaped by their cultural beliefs, knowledge, preferences, the context of diabetes clinic, access to social support and cost (Figure 1). Some examples of questions for HPs were: “What type of dietary and physical activity advice do you offer?”, “How well do know patients adhere to T2D advice?”, What do you think are some of the barriers/challenges to diabetes self-care?”, What do you think are some of the things that make self-care easy?”. Interview questions for patients included: “When it comes to what to eat, do you experience any challenges?”, “Is it difficult to follow the advice given by the HP sometimes? Interviews, which lasted between 45-55 minutes were conducted in a quite location of the hospital provided by the HPs. It provided participants with an environment to speak freely and openly about their experiences. A flexible approach to interviewing was adopted using follow-up questions, probes and comments to gain greater insight into participants’ views about diabetes self-care.^24^ Data saturation (i.e. where no new perspectives on the subject were being elicited) was achieved after conducting 33 interviews and this was confirmed with the last three study participants. Informed consent was obtained from all participants.

### Data analysis

Interviews were conducted in English and Twi (a Ghanaian vernacular), audio-recorded and transcribed verbatim. The transcripts in the Twi language were translated into English prior to analyses. Interview transcripts and field notes from observation were examined, read and re-read for data familiarization, accuracy and transcription errors were corrected. All participant identifiers were removed. All available data were analyzed manually and thematically using a directed content analysis approach, a common framework used when an existing theory can guide research questions to validate, challenge or extend such theory.^20^ Analysis was primarily deductive using a coding framework informed by the SEM, for categorising the data in relation to the key research questions about the barriers and facilitators of T2D self-care. Alongside the deductive analysis, coding was flexible with new codes being added and existing codes being deleted/modified inductively based on the data to allow new themes to emerge. Given the qualitative nature of the data findings are reported in broad terms (e.g., most, few, many, and some).

### Data credibility

Two independent qualitative researchers reviewed a random sample of six interview transcripts and field notes and all discrepancies in coding and emerging themes between the two researchers were resolved through consensus. Moreover, the researcher engaged critically with the raw data throughout the analysis process in order to gain a full understanding of what the data meant.^25^

These processes enhanced the rigor of study as whole and the credibility of its findings.

## Results

### Participant socio-demographic characteristics

Participants (patients and HPs) included a range of demographic backgrounds, as presented in Tables 1 and 2 respectively. Patients represented male (45.5%) and female (54.5%). The majority of the patients were Akan (36.3%), with monthly incomes less than GHȻ,2000 (39.5%). Moreover, most of the HPs had postgraduate education (66.7%), with work experience ranging between 3-5 years and above.

### Key themes from interviews and observation

Thematic analysis of interview transcripts and field notes revealed distinct barriers and facilitators of managing T2D across four major themes corresponding to four aspects of the SEM: 1) the counselling process and context; 2) recommended food and diet regimens; 3) social aspects of T2D self-care and 4) hopes and fears about T2D. To preserve the anonymity of study participants, verbatim quotes used to support evidence in this section are labelled in terms of “age”/ “gender” for patients or as “HP” 1, 2 or 3 for HPs.

### Perceptions of the counselling process and context

*Counselling process and context facilitators*

A key facilitator in the counselling process and context was the tendency for the HP to provide disease- and diet-specific information that help patients to assume responsibility and control over their diabetes. Thus, for instance, a typical counselling session for first-time patients starts with the dietitian educating such patients on the nature of T2D, how dietary habits influence the disease’s progression, the dietary guidelines and recommendations to be followed for managing the disease, such as losing weight, reducing sugar levels, following an individualized diet plan (i.e., a summary sheet with instructions about foods to be avoided; or those that could be taken freely, occasionally or moderately). Moreover, the dietitian often demonstrated food quantities (e.g. size of bread, meat) to be taken by patients using handy measures, such as a ladle, sardine or tomato tin; or mobile phone. The session often ended with a scheduling of the patient’s next follow-up appointment together with the requisite tests to be taken prior to this. This way, the essence of the overall counselling process is to help patients gain control over the management of the disease as HP 1 stated:

*“We tell the patient to do a lab test before the next visit, what to eat, to do some form of exercise, at least three times a week. We even tell them, how they can pack lunch to work and give other practical advice. Some patients complain a lot and I realize they don’t pay attention to the advice and are not trying their best”.*

Substantiating this view, HP 2 stated:

*“We teach the patients remedies for hypoglycemia, advise them on foods that reduce blood sugar levels and give them summary sheets of the recommendations. So, my patients really don’t have an excuse not to remember what we tell them to do”.*

Moreover, although most patients suggested that the initial diagnostic procedures undertaken by HPs, such as checking blood-sugar levels constituted sources of anxiety and fear about the disease, such tests nonetheless often served as motivators for taking the disease seriously and adopting lifestyle changes recommended by the HP, as one participant indicated:

*“Diabetes is such that you can never know you have it until you have done the test. So is it not better to know your status, than not at all, so that you can do something about it?” (Female, 59 years).*

In addition, patients reported that the rapport created by HPs through use of the local language during the care process served as a motivator for attending the clinic continually as well as adhering to treatment guidelines. Thus most patients seemed to appreciate the warm and encouraging attitude of HPs as they took the time to explain issues in their language of choice This was confirmed during observation of the care process as HPs often interacted with patients either in English or a local language (e.g. Twi, Ga-Dangme) widely spoken in Ghana.

*“The HPs here are very good. I like the way they treat us. Even if your sugar levels are high or has not improved since you last visit, they take their time to tell you what you can do to bring it down in any language at all you want…They don’t speak any big English that you can’t understand” (Male, 42 years old).*

*Counselling process and context barriers*

HPs were concerned that patients often failed to honour follow-up appointments for unknown reasons. It was only when such patients report at the hospital with a different health problem that their missed appointments were noticed.

*“When patients are diagnosed, and we give them a time to come back for review, usually after one month to see if there have been any improvements, we never see them again…And then after a long time, they show up with other conditions related to their diabetes… That is one big challenge we have” (HP 3).*

Patients also highlighted the long waiting times, that is, between one and two hours spent to see the dietician as a barrier to seeking care.

*“I sought permission from work to come here. But I have spent more than one hour without seeing anybody. Can I always be asking permission to attend the clinic?” (Male, 45 years old).*

In addition, few patients reported as a barrier, lack of acceptance of diabetes as a chronic illness as they kept thinking about it and struggled to adjust to HPs’ dietary control and regulation recommendations. Others also felt they had limited control over the condition, with others expressing denial, even a year after diagnosis and continued to have problems accepting the diagnosis. One emotional response to the lack of acceptance and control over the disease was expressed by a patient:

*“When I was diagnosed with diabetes, I was so sad and worried because my elder brother died of it. I still cry and worry a lot about this disease. The drugs they give me make me weak, so I have stopped taking them, but I know God is in control of my life” (Female, 53 years old).*

### Perceptions of recommended food and diet regimens

*Recommended food and diet regimen facilitators*

One major facilitator was HPs’ role in educating patients to make needed life-style changes regarding food intake and timing of meals as part of the strategies for managing their diabetes, as illustrated in this quote:

*“In general, the diabetic patient has to be on a low simple sugar or low simple carbohydrate diet plan. Meaning he shouldn’t take simple sugars and anything that contains sugar like soft drinks and fruit drinks. They should eat foods high in fiber regularly. We give them structured meal plans and specific meal times, they can have mid- meal snacks and we recommend foods that will not increase their blood-sugar levels. We teach them how much food they should take and an exercise regime” (HP 2).*

Moreover, HPs, being cognizant of strategies patients adopt to negotiate on dietary recommendations, often took the time to emphasize the relevance of such advice for patients as one HP stated:

*“Sometimes patients want to you to bend the dietary standards so that they can continue to eat unhealthy foods… But I stick to the regulations and spend time explaining why those unhealthy foods need to be taken out of their meal plan” (HP 1).*

*Recommended food and diet regimen barriers*

Most participants reported various barriers to diet management, including how they had to negotiate with the dietician on dietary recommendations or had to make their own decisions regarding recommended guidelines. Generally, most patients emphasized enjoying habitual Ghanaian foods (e.g., cassava, yam, plantain, cocoyam, sweet potatoes and sauces, which are rich in sugars, sodium and fat; or low in protein and fiber) that contrast with recommended dietary guidelines- and felt that they had little or no self-control when it came to dietary choices. On participant stated:

*I used to eat 8 slices of plantain and he (HP) said I should eat 4 slices. But I eat six instead because it is my favorite food. And for rice, he (HP) said I should eat 3 ladles but I eat 4 because if I eat the three I still feel hungry (Female, 53 years old).*

Regarding dietary issues another patient stated:

*Always your mind is on food, you have to be checking the time so you can eat at the right time. It is inconvenient to be carrying food with you all the time (Female, 59 years).*

Another patient described how confusing the dietician’s advice was:

*“The dietician says I should not add sugar or milk to my porridge…So it is tasteless. But I want to feel the taste…What should I do? I have stopped eating many foods and don’t know what to do again. It is all confusing” (Male, 69 years old).*

### Perceptions of the social aspects of T2D self-care 

This study found barriers or facilitators of diabetes self-care that are grouped into three categories: 1) social functions, 2) family and social ties and 3) lack of education on the social aspects of diabetes self-care during counselling sessions.

*Social functions as a barrier*

Social occasions such as weddings, funerals, naming ceremonies and birthday parties, which are very common in Ghana and embedded in strong social and cultural norms were more often described by patients as barriers than facilitators of managing T2D. Social functions were highlighted as significant barriers because there was too much temptation on offer added to a lack of healthier or more appropriate foods choices. Consequently, patients adopted various coping behaviours and practices towards social functions such as: 1) avoiding attendance by giving a flimsy excuse; 2) faking eating; or 3) asking to take the food away. One participant described her coping strategy during such gatherings:

*“These days when I go for weddings, I leave just after the main ceremony. I don’t join their food queue, everything is full of oil” (Female, 55 years old).*

Moreover, some patients avoided going out with friends by claiming they had stopped drinking as a way of maintaining a healthier lifestyle:

*“I used to take wine but now I have stopped. My friends ask me, Oh, my brother, have you become a Pastor? I don’t tell them that I have diabetes... I just give the excuse that I’m aging so I have stopped” (Male, 50 years old).*

*Family support as facilitators*

Most participants reported how family support was an important part of managing their diabetes. Some family members mentioned as supporters included spouses, children and other close and extended family members with diabetes. Most patients reported that these family members assisted with a wide range of management strategies, such as adjusting to new diet regimes and roles dictated by the patient, assisting with the preparation of recommended meals; acting as checks on patients when they were “tempted” to eat “unhealthy” foods or ate late - and serving as reminders to the check their blood-sugar levels. One participant stated:

*“I think my husband and children are very supportive. I rely on them a great deal to manage my diabetes… Sometimes my eldest son watching me getting ready to have porridge for breakfast can quickly alert me by saying… “Mama you’ve added one teaspoon of sugar already…that’s enough!! The doctor said you shouldn’t take sugar at all” (Female, 49 years old).*

*Family support as a barrier*

Family members sometimes constituted barriers for patients managing the condition as they insisted on eating their regular meals, rather than adjusting to the needs of the diabetic patient as one participant stated:

*“I have been trying to stop eating white rice, and to eat brown rice instead to reduce my high sugar levels, but my household does not like the brown rice” (Female, 41 years old*).

*Limited education on social aspects of T2D self-care as a barrier*

Evidence from counselling sessions revealed that providers often focussed essentially on helping the patient to manage the disease clinically, in terms of diet, physical activity and glycemic control among others, with limited emphasis on the social dimensions of the disease, such as how to disclose it to family members or mobilise social support from such family members for managing various aspects of the disease. This could serve as a significant hindrance to patients managing the disease on a day-to-day basis.

### Patients’ hopes and fears about T2D 

*Patients’ hopes about diabetes as facilitator*

As, a facilitator, some patients hoped for a cure bio-medically and through faith in God concurrently, as one participant stated:

*“I was worried when I was diagnosed because my sister-in-law died of it (diabetes)…so when they told me I had the disease, I was afraid and I prayed to God for healing. I believe God heard my prayers because when I came today the lab man told me my sugar level was ok” (Female, 53 years old).*

*Patients’ fears about diabetes as barriers*

First, patients expressed fears about how their diabetes might adversely affect family members in two major ways that impinges on effective self-management: 1) fear of becoming a burden on the family; and 2) fear of not obtaining the needed support from family members. Moreover, patients reported fears about complications that could result from diabetes, such as, general malaise, ulceric wounds, retinopathy, renal damage, heart disease and stroke as significant barriers to self-management. In addition, while most male participants expressed worry about how diabetes could adversely affect their sexual drive, female participants were worrisome about how it could lead to infertility in women. In order to allay some of these fears, some patients prefer not to disclose their diabetic status to family members as one participant stated:

*“I did not let my wife know I am diabetic. I don’t know how she’s going to take the message. But I think with time, I will let her know” (Male, 50 years old).*

In contrast, some patients pretend to be well, despite a diagnoses, as ways of managing prevailing fears about the disease: 

*“Even though I am feeling sad about my diagnosis, I don’t want my husband and children to be worried. I have to be strong for them, so I just tell them I am okay, although I know very well, I’m not to feeling well” (Female, 45 years old).*

## Discussion

This study aimed to explore the barriers and facilitators of T2D self-management among adult patients attending a specialist diabetes clinic in Accra, Ghana. Findings from observations, patients’ and HPs’ perspectives were similar to prior studies regarding diabetes management^26^ specifically, and chronic conditions more generally.^27^ The findings highlighted not only facilitators and barriers to T2D management during the counseling process and contexts of providing care, but also- related recommend food and diet regimens; social factors and patients’ hopes and fears about the disease. The findings support other studies that have attempted to understand and address the challenges of working within adult diabetic populations.^28-30^ Moreover, while most studies in Ghana to date have focused essentially on factors such as patients’ demographic characteristics (e.g., age, gender, education, and religion) diet and body weight that are associated with adherence to recommended self-care behaviours^17,18^; few qualitative studies have been undertaken to explore the multiple facilitators and barriers pertinent to the self-care of T2D. This study therefore adds to the rather sparse literature in this area. Although the facilitators and barriers to care more generally, have been discussed in the literature, what became clear in the data analysis was the extent to which diabetes care was dependent on the dynamic interaction between factors associated with the counselling process and context, recommended food and diet regimens, social context of managing the disease; as well as the hopes and fears about the disease as described next.

Regarding the counselling process and context, key facilitators included HPs’ role in helping patients to assume responsibility and control over their diabetes, patient acceptance of the diagnosis and rapport. Patient education, acceptance of diagnosis and motivation to engage in self-management in the context of patient-provider relationship have been cited previously.^14,31^ However, in order to effect the most beneficial lifestyle changes, patients must be actively involved as experts and health care providers as advisors during the care and counselling process,^32^ similar to this study’s findings.

Rapport has been increasingly recognized as an important facilitator of T2D self-care.^33,34^ Patients reported that HPs’ use of the local language in diabetes education facilitated trust and rapport in their relationship and motivated continued attendance at the clinic as well as compliance within care regimens. This finding implies that language barrier could hamper effective T2D self-management as some authors have suggested^34^ and hence, tailoring diabetes interventions according to the language of the patient, especially in multi-ethnic societies, such as Ghana and other SSA countries can contribute to more effective self-management behaviours and practices.^35^

Moreover, HPs reported barriers of patients not keeping follow-up appointments, while patients identified long waiting times and lack of acceptance of diagnosis as barriers. The tendency for patients to miss follow-up visits could be attributed to time constraints, lack of symptoms, long waiting times and high cost of consultation as reported in other studies.^36,37^ The issue of cost of consultation is probably due to the fact that patients must pay out-of-pocket because the private specialist hospital of this study is not a registered service provider of Ghana’s National Health Insurance Scheme established to provide universal health coverage for the citizenry. These findings suggest that interventions to improve follow-up visits need to focus on educating patients about the importance of regular check-ups as well as other strategies designed to tackle multiple barriers simultaneously (e.g., to reduce out-of-pocket payments, quicker consultations), may be more effective in reducing non-attendance than strategies that target one barrier.^26^ In addition, the few patients reporting a lack of acceptance of their diabetes, and hence lack of motivation to engage in self-care identified as a barrier, has been associated with the disease’s asymptomatic nature, perceived seriousness and fears of potential death resulting from the condition.^38^ Previous studies also highlight the strong emotional distress and depression recognizable in diabetic patients that poses a significant barrier to self-care,^39^ and for patients in this study, treatment might be complicated by limited access to mental health services - that is characteristic of low resources settings, including most SSA countries.^40^ Some diabetes self-care programs have been shown to be effective in decreasing diabetes-related anxiety and distress in disadvantaged populations, and might be models to replicate in low resource settings.^41^ In relation to recommended food and diet regimens, barriers to lifestyle change received more prominence in patients’ accounts than facilitating factors. Mostly, patients’ concerns were about changing well-established habits, a general dislike of many aspects of their recommended diets and confusion about how to deal with particular aspects of the diet-factors which have been documented in previous studies.^28,42^ Although, most patients in this study seemed to have some broad knowledge about the importance of diet in diabetes management, they appeared to be ill-equipped with specific information regarding recommended diets (e.g., acceptable types, quantities or amounts) that could help them to effect needed lifestyle changes. This lack of knowledge thus constituted a significant source of frustration for most patients trying to adjust to new diet regimens. While knowledge acquisition by patients during the diabetes care process is considered important for effective self-management, available evidence suggests that such knowledge might not be consistently applied in practice^43^ as this study found. Increasingly, it has been suggested that tailored guidance based on individual needs and the provision of diet-specific information, previously shown to be effective in patients’ goal setting behaviors and habit modification practices should be the central focus of diabetes counselling.^44^

Concerning social functions, most participants were of the perception that such occasions interfered with dietary alterations needed for effective diabetes care. This often resulted in patients reducing or avoiding some interactions with family and friends involving food and drinks. This means that patients in this study found a more positive disposition towards problem-solving and more rational decision-making towards controlling their diabetes. The exhibit of resiliency in diabetes care, such as found in this study, is consistent with other studies in New England and Iowa (US) among diabetes patients, in which those in better control of the disease; a) took diabetes more seriously; b) knew diet management was important; and c) viewed lifestyle changes as central to controlling diabetes.^34,45^ This finding also supports another qualitative study in Germany, which found that people with T2D often experience an intense moral pressure to give up many pleasures and live a life of abstinence from certain foods in order to better control the disease - and suggest that taking into account this moral pressure, and patients’ personal efforts and strategies to establish healthy behaviours, might help to build better and trusting patient-provider relationships.^46^

Moreover, patients’ family and social ties either facilitated or hindered self-care; for example, some family members assisted the patient with preparing recommend meals, while others diverted the patient from their goals regarding diet. The importance of family and friends as facilitators or barriers has been reported in previous research.^14,31^ This implies that rather than simply focusing on technical issues, such as diet management, physical activity and glycemic control during the counselling process, providers could also educate patients on the role of family members and friends in shaping self-management. This is particularly important in Ghanaian and other African societies where strong emphasis is placed on family relationships as an enduring cultural value. However, this study, found limited focus on the role of such family interactions in diabetes-self-care during patient counselling sessions. The reason for this tendency is not clear. It might be because providers were often overwhelmed by the large number of patients attending the diabetes clinic in a particular day, and as such, tended to focus more on the technical aspects of managing the disease to the exclusion of its social dimensions. Greater clarity is needed to guide action.

Patients’ hopes and fears as positive or negative emotional responses to the diagnosis and as barriers to or facilitators of on-going self-management of T2D have been increasingly recognized.^47,48^ A finding was that patients relied on a combination of bio-medicine, religion and belief in God to help with diabetes self-management. This is consistent with other literature on diabetes self-care among African Americans.^12^ Other research reinforces the importance of religion and belief in God for diabetes management especially, in medically pluralistic health care settings, including SSA countries, where people engage in “health shopping” (i.e., using different medical systems either concurrently or successively) in times of ill-health.^49^ Importantly, these studies have suggested that faith in God can be a major source of support as well as a major barrier in diabetes self-care. In this study, fear of diabetes made it difficult for patients to disclose the diagnosis to family members and/or pretended to be well, and adhere to dietary guidelines and medications more generally, in the belief that God would take care of them, such that it was not necessary to worry about their diabetes. This signifies a sense of fatalism, which has been associated with diabetes self-management among African Americans, suggesting that the diabetes was beyond their control.^50^ This finding reinforces a range of strong emotional responses (e.g., fear, sadness, anxiety, depression) and hopes associated religious beliefs and practices influencing self-management that HPs should consider as part of providing patient-centered care for better outcomes.

### Limitations and future research 

This study suffers one major limitation, that is, it did not explore T2D self-care behaviours and practices of patients outside the clinic (e.g., during meal preparation at home; at work places during lunch breaks) to observe meal availability and eating routines; and as well, during shopping tours to observe food availability and shopping practices. These environments provide additional contexts for more or less effective management of the disease. Future research is needed to provide greater insights into patients’ self-care behaviours and practices in such contexts.

## Conclusion

Adults with T2D face many inter-related challenges to diabetes self-care than enabling factors around four major themes: These are: 1) the counselling process and context (patients missing follow-up appointments, unacceptance of diagnosis); 2) recommended food and diet regimens (changing longstanding dietary habits, dislike and confusion about recommended diets); 3) social aspects (social functions interfering with recommended dietary regimens, family members diverting patient from dietary goals) and 4) fears (non-disclosure to family member/pretense of being well). Integrated self-management interventions are needed to address these barriers. These include tailoring dietary education to specific needs of the patient, guiding patients on how to manage their diabetes during social occasions and among family members and friends; and providing mental health support for handling strong emotional reactions to the disease.

## Ethical approval

Ethical clearance (RPN005/CSIR-IRB/2014) was obtained from the Institutional Review Board of the Council for Scientific and Industrial Research in Accra, Ghana. Informed consent was obtained from all study participants.

## Competing interests

None declared.

## Funding

No funding was received for the study.

## Author’s contribution

MH conceived the study, collected, analyzed and interpreted the data; and drafted the manuscript.

## Acknowledgments

The author thanks the study participants for their time and support.

**Table 1:** Selected characteristics of patients (n=33)

**Variable**	**No.**	**%**
Gender		
Male	15	45.5
Female	18	54.5
Ethnicity		
Ga-Dangme	9	27.3
Akan	12	36.3
Ewe	8	24.2
Dagbani	2	6.1
Others	2	6.1
Educational level		
Basic (11 years)	14	42.4
Secondary (14 years)	10	30.3
Tertiary (18 years)	9	27.3
Occupation		
Government employee	16	48.5
Privately employed	9	27.3
Others	8	24.2
Monthly income (in Ghana cedis)		
˂ GHȻ 2000	13	39.4
GHȻ 2001–3000	12	36.4
˃ GHȻ 3000	8	24.2

**Table 2:** Selected characteristics of health providers (n=3)

**Variable**	**No.**	**%**
Gender		
Male	2	66.7
Female	1	33.3
Educational level		
Undergraduate	1	33.3
Postgraduate	2	66.7
Years of experience		
1 Year	1	33.3
3 Years	1	33.3
Above 5 years	1	33.3
